# Heat Shock-Induced Three-Dimensional-Like Proliferation of Normal Human Fibroblasts Mediated by Pressed Silk

**DOI:** 10.3390/ijms10114963

**Published:** 2009-11-12

**Authors:** Fukumi Hiragami, Hirotoshi Motoda, Toshiaki Takezawa, Chiyuki Takabayashi, Shigeki Inoue, Yuji Wakatake, Yoshio Kano

**Affiliations:** 1 Department of Physical Therapy, School of Health Science, Kibi International University, 8 Iga-machi Takahashi City, Okayama 716-8505, Japan; E-Mails: hmotoda@kiui.ac.jp (H.M.); shigeki025877@yahoo.co.jp (S.I.); mayw1230_rpt_com@yahoo.co.jp (Y.W.); 2 Laboratory of Animal Cell Biology, National Institute of Agrobiological Sciences, Ikenodai 2, Tsukuba, Ibaraki 305-0901, Japan; E-Mail: ttakezaw@nias.affrc.go.jp; 3 Laboratory of New Silk Materials, National Institute of Agrobiological Sciences, Gouda 1-4-8, Okaya, Nagano 394-0021, Japan; E-Mail: ctaka@nias.affrc.go.jp; 4 Department of Occupational Therapy, School of Health Science, Kibi International University, 8 Iga-machi Takahashi City, Okayama 716-8505, Japan; E-Mail: yoshio@kiui.ac.jp

**Keywords:** normal human dermal fibroblasts, heat treatment, three-dimensional-like proliferation patterns, p38 MAPK, Hsp27

## Abstract

The aim of this study was to determine the optimal heat treatment conditions for enhancement of pressed silk-mediated 3D-like proliferation of normal human dermal fibroblasts, as well as to determine the responses to heat shock of cells and intracellular signaling pathways. The beginning of 3D-like pattern formation of cells was observed in the second week after the start of the experiment. The mean rates of beginning of 3D-like pattern formation by cells heat-treated at 40 ºC and 43 ºC for 10 min were significantly higher (3.2- and 8.6-fold, respectively) than that of untreated cells. We found that apoptosis had occurred in 7.5% and 50.0% of the cells at one week after heat treatment for 10 min at 43 ºC and 45 ºC, respectively. Western blot analysis demonstrated that phosphorylation of p38 MAPK and that of Hsp27 were markedly increased by heat treatment at 43 ºC for 10 min. The results of an experiment using a p38 MAPK inhibitor and Hsp27 inhibitor suggest that activation of p38 MAPK by heat shock is associated with 3D-like cell proliferation and that Hsp27 contributes to the inhibition of apoptosis. The results of this study should be useful for further studies aimed at elucidation of the physiologic mechanisms underlying thermotherapy.

## 1. Introduction

Physical therapy employing electrical stimulation and ultrasound/ultraviolet or laser irradiation is widely used for wound healing of pressure sores [1,2]. Infrared therapy, one type of thermotherapy, is more effective for skin ulcers and pressure sores [3]. Experiments using cells subjected to various heat conditions are important for elucidating the mechanism of wound healing and for determining optimal treatment temperature and treatment time in order to maximize the positive effects of thermotherapy.

When thermotherapy is carried out, heat treatment enhances the proliferation of connective tissue cells. However, the heat treatment used in hyperthermia induces apoptosis of cancer cells [4]. Thermotherapy is different from hyperthermia used for heat treatment of a tumor. This means that the determination of optimal heat conditions is important. Thus, these findings suggest that it is important to determine the optimal heat treatment condition in which thermotherapy is effective as well as to determine the optimal heat treatment for hyperthermia [4].

Cell biology studies on the effects of heat treatment have been conducted in recent years, and have indicated that heat treatment may have roles in both cell proliferation and cell death [5]. It has already been shown that cells respond to exposure to elevated temperatures by increasing production of heat shock proteins (Hsp) such as Hsp70, Hsp27 and Akt [6]. p38 MAPK, a member of the family of mitogen-activated protein kinases (MAPKs), is essential for the triggering of heat shock protein expression by heat stress [7].

In our study, C3H10T1/2 mouse fibroblasts were cultured with HA (hydroxyapatite) granules for 10 weeks after heat treatment at 40–44 ºC or 45 ºC for 2–360 min [8]. Then optimal conditions of temperature of heat treatment for induction of three-dimensional (3D)-like proliferation of cells were determined. The optimal conditions of heat treatment to induce 3D-like proliferation of cells were 43 ºC for 10 min. We have recently investigated the role of the p38MAPK pathway in heat shock-induced 3D-like cell proliferation [9].

HA shows a high level of bioactivity and is used in a clinical setting in the dental and orthopedic fields. Additionally, silk fiber has good mechanical strength, good stability in the living body and good molderability and is used for surgical sutures. We first compared the difference in 3D-like proliferation ability of the two different materials, HA and pressed silk. In a previous study, we investigated the 3D-like proliferation pattern of mouse fibroblasts induced by heat treatment. In the present study, we observed the changes in the 3D-like proliferation pattern induced by heat treatment using normal human dermal fibroblasts (NHDFs). We also examined the effects of responses of cells to heat treatment on intracellular signaling systems of p38 MAPK and Hsp27.

The aim of the present study was to determine the optimal heat-treatment conditions for enhancement of pressed silk-mediated 3D-like proliferation of NHDFs.

## 2. Results

### 2.1. 3D-like proliferation of cells subjected to heat treatment

**Figure 1.**
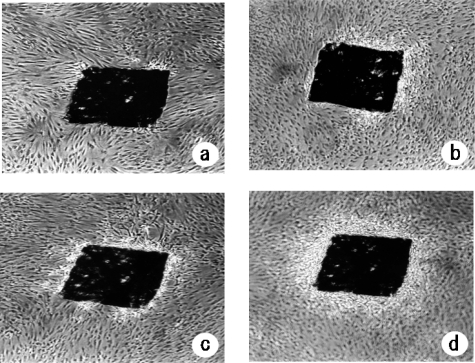
Formation of a 3D-like proliferation pattern of NHDFs mediated by pressed silk. Upper panels (a, b): Confluent cultures of NHDFs were trypsinized and seeded into flasks with pressed silk under the conditions of heat treatment at 43 ºC for 10 min and cultured for one week (a) or two weeks (b). Lower panels (c, d): Phase-contrast photomicrographs of cells that had been heat-treated at 43 ºC for 10 min after two weeks (c) and three weeks (d) of culture with pressed silk (original magnification, ×40).

Patterns of cell proliferation were observed over a period of five weeks. No formation of a 3D-like pattern of cell proliferation was observed in the cultures, but actively proliferating cells had already surrounded pressed silk at one week after heat treatment at 43 ºC for 10 min. Similarly, in the first week after heat treatment at 43 ºC for 10 min, some signs of 3D-like pattern formation were seen. In the second week after heat treatment, the layers of cells that had formed around the pressed silk had begun to form or had almost completely formed a 3D-like pattern. In the third week after heat treatment, some complete formation of a 3D-like pattern was seen. It was found that the 3D-like proliferation of cells changed in stages from “signs of 3D-like pattern formation”, “beginning of 3D-like pattern formation”, “almost complete formation” and finally “complete formation” (Figure 1).

### 2.2. Process and rates of formation of 3D-like cell proliferation pattern

**Figure 2.**
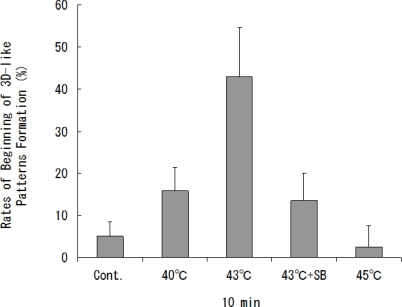
Mean rates of beginning of 3D-like pattern formation (percentage of five consecutive trials) by cells treated at 40 ºC and 43 ºC for 10 min, 43 ºC for 10 min in the presence of the p38 MAPK inhibitor SB203580 (2 μM), or 45 ºC for 10 min and by cells not heat-treated (as a control group). The rates for cells that had been treated at 40 ºC for 10 min and at 43 ºC for 10 min after two weeks were significantly higher than the rate for untreated cells (*p < 0.05).

Significant changes in the heat-treated cells, including those around pressed silk, were observed until the second week after the start of the experiment. The beginning of 3D-like pattern formation occurred when cells were heat-treated at 40 ºC or 43 ºC for 10 min and cultured for two weeks. In the second week after the start of the experiment, the mean rates of the beginning of 3D-like pattern formation were 5.0 ± 3.4% for untreated cells, 15.8 ± 5.5% for cells heat-treated at 40 ºC for 10 min, 43.0 ± 11.6% for cells heat-treated at 43 ºC for 10 min, 13.5 ± 6.5% for cells heat-treated at 43 ºC for 10 min with SB203580, and 2.5 ± 5.0% for cells heat-treated at 45 ºC for 10 min (Figure 2). The mean rate of beginning of 3D-like pattern formation for cells heat-treated at 45 ºC 10 min was 0.5-fold lower than that for untreated cells, but the mean rates of formation of 3D-like proliferation patterns by cells heat-treated at 40 ºC for 10 min and at 43 ºC for 10 min were significantly higher (3.2- and 8.6-fold higher, respectively) than that by untreated cells (*p* < 0.05).

### 2.3. Stimulation of DNA synthesis by heat treatment

**Figure 3.**
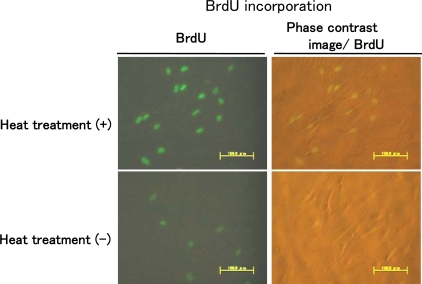
Stimulation of DNA synthesis by heat treatment. NHDF cells were plated on sterile glass coverslips coated with poly-D-lysine and were heat-treated or not heat-treated and then pulsed for 60 min with 10 μM BrdU. Immunostaining was performed using an anti-BrdU kit and was observed using a fluorescent microscope (200×).

Cells were plated on sterile glass coverslips and they were heat-treated at 43 ºC for 10 min after allowing attachment of the cells for 4 h and then 24 h later were pulsed for 60 min with 10 μM BrdU. Quantification of nuclei that incorporated BrdU upon heat treatment revealed that heat treatment resulted in an ability to block BrdU incorporation, indicating that heat treatment led to induction of DNA synthesis (Figure 3). The stimulatory effect of short heat treatment on DNA synthesis was much greater in heat-treated cells than in control that had not been heat-treated.

### 2.4. Survival curve of heat-treated NHDFs

**Figure 4.**
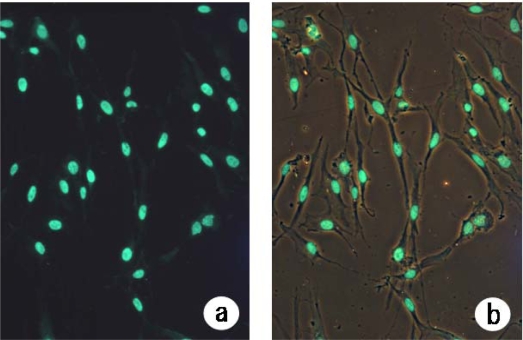
Morphology of intact cells and nuclear morphology of apoptotic cells. Chamber slide culture cells that had been incubated for 3 days following heat treatment at 45 ºC for 20 min displayed apoptotic morphologies. The morphology of these cells was determined by TUNEL staining and fluorescence microscopic observation and was shown in Fluorescein (a) and Fluorescein/Phase contrast image (b) (×200).

Cells exhibiting apoptotic morphology and intact cells observed in chamber-slide culture after 3 days of incubation following heat treatment at 45 ºC for 20 min are shown in Figure 4; 100% of all cells exhibited apoptotic morphology.

**Figure 5.**
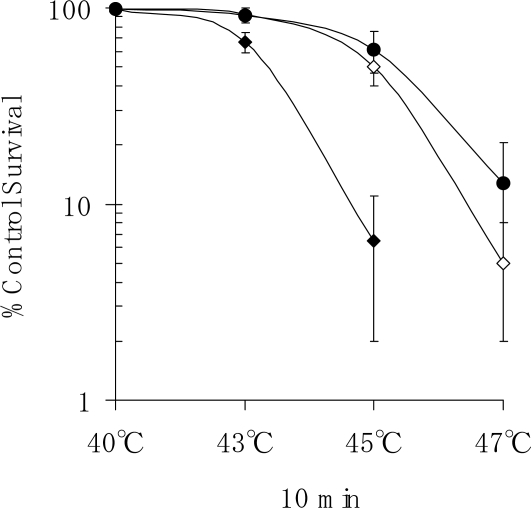
Survival curve of heat-treated NHDFs. Cells were trypsinized and plated in triplicated in 60-mm-diameter Corning dishes. After allowing attachment of the cells for 4 h or after allowing culture of cells for 24 h (•), the cells were heat-treated at 40, 43, 45 or 47 ºC for 10 min in the presence (♦) or absence (⋄) of the Hsp inhibitor KNK437 at 3.6 μM. The heat-treated cells were incubated for 10 days until colony staining. Each value is the mean ± S.E.M. for five independent experiments.

Figure 5 shows the survival curve of heat-treated NHDFs. The cells were exposed to temperatures of 40, 43, 45 or 47 ºC for 10 min and then incubated for 10 days to determine colony-forming ability. The results from three separate experiments showed that almost 50.0% of the cells that had been heat-treated at 45 ºC for 10 min underwent apoptosis in the first week after treatment, while less than 7.5% of the cells that had been heat-treated at 43 ºC for 10 min underwent apoptosis in the first week after treatment. However, there was little cell death in cells that had been trypsinized and plated in dishes and then heat-treated at 45 °C for 10 min after culture for 24 h. Moreover, treatment with the Hsp inhibitor KNK437 stimulated cell death induced by heat shock treatment.

### 2.5. Activation of p38 MAPK and Hsp27

**Figure 6.**
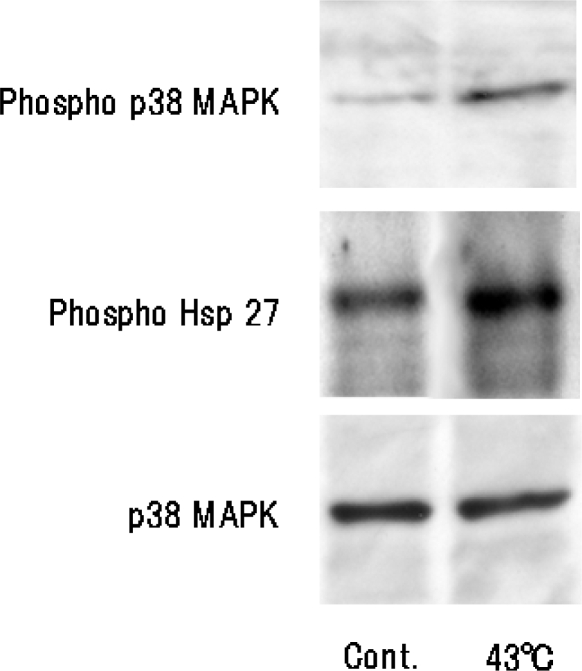
Heat shock-induced activation of p38 MAPK and Hsp27 in NHDFs. Cells were serum-starved and subjected to heat shock at 43 ºC for 10 min or not exposed to heat shock (control). After treatment, cells were lysed, and protein extracts were analyzed by Western blotting using antibodies specific for phospho-Hsp27 and phospho-p38 MAPK.

We investigated the heat shock-induced activation of Hsp27 and p38 MAPK in NHDFs by Western blot analysis. We found that both p38 MAPK and Hsp27 were significantly activated by heat shock at 43 ºC for 10 min in NHDFs (Figure 6). These findings indicated that the p38 MAPK pathway plays a key role in pressed silk-mediated 3D-like proliferation and that Hsp27 contributes to the inhibition of apoptosis of NHDFs by heat treatment.

## 3. Discussion

The aim of our previous study was to determine the optimal condition under which heat treatment is effective for enhancing 3D-like cell proliferation [8,9]. C3H10T1/2 mouse fibroblasts were cultured with HA granules for 10 weeks after optimal heat-treatment conditions. The formation of a 3D-like cell proliferation pattern was observed from the fifth week after heat treatment under optimal conditions. Therefore, the results of our previous study should be useful for elucidation of the physiologic mechanisms underlying thermotherapy.

In the present study, we investigated the changes in proliferation pattern of NHDFs induced by heat treatment using pressed silk. The formation of a 3D-like cell proliferation pattern was observed form the third week after optimal heat treatment under optimal conditions. Thus, a 3D-like proliferation pattern of NHDFs was formed more rapidly than that observed in mouse fibroblasts. Armour’s report indicates that heat shock does not normally induce extra cell proliferation [10]. However, when cells were heat-treated at 43 ºC for 10 min after allowing attachment of the cells for 4 h, heat treatment led to induction of DNA synthesis (Figure 3). These results indicate that heat shock may induce extra cell proliferation.

Both our previous study and the present study showed that the optimal conditions of heat treatment to induce 3D-like cell proliferation were 43 ºC for 10 min [8]. In the present study, the mean rate of beginning of 3D-like pattern formation for cells heat-treated at 43 ºC for 10 min after two weeks was 43.0 ± 11.6% in an experiment using mixed cultures of NHDFs and pressed silk. However, the mean rate after two weeks in an experiment using mixed cultures of mouse fibroblasts and pressed silk was 26.3 ± 6.5% (data not shown). Moreover, in an experiment in our previous study using mixed cultures of mouse fibroblasts and HA, no formation of a 3D-like pattern was observed at two weeks, but the mean rate at three weeks was 15.2 ± 3.4%. Therefore, the results for beginning of 3D-like pattern formation of cells should be useful for elucidation of the physiologic mechanisms underlying thermotherapy.

HA has been widely used as a substitute material for bone because it can be bonded directly to living bone and teeth. HA [chemical formula Ca_10_ (PO_4_)_6_(OH)_2_] ceramic is compact, with multinuclear granules that have continuous pores ranging from 30 to 200 microns in diameter [1,3]. On the other hand, silk fiber has recently been studied as a scaffold for tissue engineering because of its excellent biocompatibility and bioabsorbability and its low level of inflammatory potential [15–18]. A pressed silk sheet composed of cocoon filaments is a valuable scaffold that can facilitate 3D culture of fibroblasts [11]. More NHDFs adhered to the pressed silk than to HA, and a 3D-like proliferation pattern was formed more rapidly on pressed silk than on HA. These results demonstrated that a 3D-like proliferation pattern of cells was composed by two different materials, inorganic HA or organic pressed silk. The scaffold enabled culturing of anchorage-dependent cells on surfaces of the pressed silk resulted in reconstruction of a 3D culture. Therefore, a 3D-like proliferation pattern was formed more rapidly on pressed silk than on HA.

In the present study, we also conducted an experiment on the survival of human fibroblasts subjected to heat shock. We found that apoptosis had occurred in 50.0% of cells at one week after heat treatment at 45 ºC for 10 min (Figure 5). Staining of cells that had been heat-treated at 45 ºC for 20 min revealed that apoptosis had occurred in many of those cells at three days after the start of culture (Figure 4). It was found that apoptosis had occurred in 7.5% of cells in the first week after heat treatment at 43 ºC for 10 min. Our results indicate that heat treatment may stimulate cell proliferation, leading to the repair of damaged tissue. It is thought that the results of the present study provide important information for determining the appropriate heat conditions for thermotherapy. The formation of a 3D-like proliferation pattern around pressed silk is the result of change in the cell membrane caused by heat shock when it has reached the threshold and a subsequent induction of gene expression to synthesize a large amount of extracellular matrix.

The optimal condition for heat treatment to induce 3D-like cell proliferation was 43 ºC for 10 min (Figure 2). Heat treatment of cells for 10 min at 43 ºC will result in some cell damage, but the cells will later recover with enhanced functions. It has already been shown that stress-activated p38 MAPK and stress-responsive Hsp27 are two important proteins involved in cellular processes responding to extracellular stimuli and various stress. Therefore, we conducted an experiment on heat shock-induced formation of a 3D-like cell proliferation pattern, and we also examined the effects of responses of cells to heat treatment on intracellular signaling systems of p38 MAPK and Hsp27. Heat shock-induced 3D-like pattern formation was strongly inhibited by heat treatment at 43°C for 10 min with the p38 MAPK inhibitor SB203580. However, there was little cell death in cells that had been trypsinized and plated in dishes and then heat-treated at 45 °C for 10 min after culture for 24 h. Western blot analysis demonstrated that activation of p38 MAPK and activation of Hsp27 were markedly enhanced by heat treatment at 43 ºC for 10 min (Figure 6). Also, the survival rate of NHDFs subjected to heat shock was greatly reduced by treatment with the Hsp27 inhibitor KNK437 (Figure 5). Therefore, we concluded that 3D-like proliferation of heat-treated cells was induced by activation of p38 MAPK and Hsp27.

We performed experiments using BrdU labeling analysis, and the results are shown in Figure 3. In our previous study, we found that heat-shock-induced formation of a 3D-like proliferation pattern was strongly inhibited by treatment with the p38 MAPK inhibitor SB203580, and we concluded that 3D-like proliferation of heat-treated cells was induced by activation of p38 MAPK, but not ERK [8]. Also, in our study, we investigated whether the p38 MAPK pathway inhibitor SB203580, but not the ERK pathway blocker UO126, inhibited the ability of PC12m3 and PC23m32 cells to induce neurite outgrowth in response to osmotic shock, and we found that expression of a nonactivatable form of p38, but not that of wild-type p38, significantly blocked neurite outgrowth induced by osmotic shock [19]. Our recent study indicated that p38 MAPK is associated with differentiation and that JNK contributes to the induction of apoptosis [20].

We also observed cell proliferation and increasing production of HSP after allowing attachment of the cells following heat treatment at 43 °C for 10 min. We also observed DNA synthesis following heat treatment at 43 °C for 10 min. These results are associated with the “hormesis” theory of pattern. Rattan *et al*. [21] have shown hormetic effects of a mild heat stress regime involving exposure of serially passaged human skin fibroblasts to a temperature of 41 °C for 1 h twice a week throughout their replicative lifespan *in vitro*, and they reported a variety of beneficial effects, such as maintenance of youthful cellular morphology, enhancement of replicative lifespan and enhancement of proteasome activity. It is notable that several age-related alterations, such as alterations in levels of various heat shock proteins (HSP), [22] were affected by repeated mild heat shock. In the future, we have planning heat shock-induced three-dimensional-like proliferation of normal human fibroblasts mediated by pressed silk using aged cells (over 40 population doublings).

## 4. Conclusions

When culture of NHDFs was exposed to heat stress at 43 ºC for 10 min, intense activity of p38 MAPK was observed, and activation of Hsp27 was relatively intense in the same heat condition. These findings indicate that p38 MAPK is associated with 3D-like proliferation and that Hsp27 contributes to the inhibition of apoptosis. Our findings suggest that it is important to determine the optimal heat treatment condition in which thermotherapy is effective. The results of this study should be useful for further studies aimed at elucidation of the cellular mechanisms underlying the therapeutic effects of heat.

## 5. Experimental Section

### 5.1. Cells and culture conditions

All experiments were carried out with a normal human fibroblast strain (NHDFs) designated KF-4001. This strain was obtained from Kurabo Industries (Osaka, Japan) at passage 1; it was originally derived from the foreskin of a normal newborn male. KF-4001 cells at four passages (eight population doublings) were used in the experiments. Life span was measured in terms of total population doublings after the cultures had been established. Fibroblast outgrowths from several small pieces (1 mm^3^) of skin explant were considered as passage “zero.” We considered passage four to be at eight population doublings as judged by the fact that one passage represents approximately two population doublings in cultures passaged by a 1:4 split regimen. KF-4001 cells were subcultured in Dulbecco’s modified Eagle’s medium (DMEM) containing 10% heat-inactivated fetal bovine serum (FBS), 20 mM *N*-2-hydroxyethylpiperadine-*N’*-2-ethanesulfonic acid (HEPES), penicillin (50 U/mL), and streptomycin (50 *μ*g/mL). The cells were grown at 37 ºC in a humidified incubator with 5% CO_2_/95% air in a plastic flask (Falcon; BD Biosciences Discovery Labware, Lincoln Park, NJ, USA). The culture medium was changed every 3 days. Subcultivation was performed whenever the cultures became confluent (approximately 4 × 10^5^ cells/25 cm^2^ of the flask at confluence) by adding 0.25% trypsin solution and allowing the cultures to stand at room temperature until they began to dislodge themselves from the flasks, at which time they were suspended in fresh culture medium and dispensed as quickly as possible into new flasks at a 1:4 dilution. Using the Hoechst 33258 (Polyscience, Warrington, PA, USA) staining method, the cultures were tested at several intervals for mycoplasma contamination and found to be negative for contamination.

### 5.2. Preparation of pressed silks

The pressed silks were randomly distributed in the flasks. Pressed silk that consists of a network structure of numerous cocoon filaments each about 15 μm in diameter and with pore size axes shorter than 200 μm has recently been developed [11]. Cocoon filaments spread uniformly on a pressing machine were moistened and then pressed at a high temperature of 120 ºC to reagglutinate a protein of sericin covering a core protein of fibroin in order to yield a pressed silk sheet. The sheet had a thickness of 216 μm. On the other hand, cells cultured on the scaffold did not efficiently invade into the interstices of the silk fibers. Fibroblasts on a pressed silk sheet coated with type-I collagen were able to form a 3D connective tissue model to promote cellular collagen synthesis [11]. However, in the present study, we used a pressed silk sheet without coating of type-I collagen. The pressed silk used in this experiment was cut into square shapes (0.5 mm × 0.5 mm). One hundred sheets of pressed silk were put into each test tube before the experiment. The tubes containing pressed silks were sterilized in 70% ethanol for 2 weeks and washed in 100% ethanol and then dried.

### 5.3. Heat treatment of cells

We conducted an experiment to observe patterns of proliferation of cells that had been subjected to heat shock treatment and patterns of proliferation of cells that had not been subjected to heat shock treatment. Heat shock treatment was performed in a water bath (Water Bath Shaker Personal 11; Yamato, Tokyo, Japan). Confluent cultures of NHDFs were trypsinized and seeded into flasks that each contained 100 pressed silk sheets. For heat shock treatment, the flasks were closed, and the screw area was tightly sealed with wax paper. The flasks were then completely immersed in a water bath. Then the cells were heat-treated at 40, 43 and 45 ºC for 10 min by using the water bath. The temperature was measured using a spot thermometer (TA-0510; Minolta Camera Co. Ltd., Tokyo, Japan) with a precision of ±0.1 ºC. The medium in the flask reached 43 ºC within one minute of immersion. After heating, the screws were loosened, and the cultures were kept in a CO_2_ incubator for five weeks without stimulation after heat shock treatment.

### 5.4. 3D-like proliferation of cells

Formation of 3D-like proliferation patterns surrounding the pressed silk was observed under a phase contrast microscope (Handstand Microscope TMS-F, ×40; Nikon Co. Ltd., Tokyo, Japan) after heat shock treatment for 3 weeks with or without the p38 MAPK inhibitor SB203580 [4-(4-fluorophenyl)-2-(4-methylsulfinylphenyl)-5-(4-pyridyl)imidazole], which was obtained from Sigma-Aldrich (St. Louis, MO, USA). A structure was considered to be the beginning of 3D-like pattern formation when the layer of cells surrounded more than half and less than three quarters of the pressed silk. The beginning of 3D-like pattern formation around all of the pressed silk sheets was examined. The frequency of the beginning of 3D-like pattern formation was determined by counting the number of pressed silk sheets in each flask (containing 100 pressed silk sheets).

### 5.5. Immunocytochemistry

For the experiments using NHDF cells, BrdU was purchased from Sigma-Aldrich and used according to a previously published protocol [23]. Cells were plated on sterile glass coverslips coated with poly-D-lysine and were heat-treated at 43 ºC for 10 min and then 24 h later were pulsed for 60 min with 10 μM BrdU before fixation with 4% PAF in PBS. Immunostaining was performed as suggested by the manufacturer of the in situ cell proliferation kit (Roche). Scans of stained cells were made using a fluorescent microscope (mode 1 ×70; Olympus).

### 5.6. Survival assay

Survival fractions were determined by a standard colony-formation assay. Plastic Corning dishes of 60 mm in diameter were seeded with an appropriate number of cells such that, accounting for the cloning efficiency and toxicity of the particular treatment, 50–100 macroscopic colonies would have developed when the cultures were fixed and stained approx. 10 days later. The NHDFs were exposed to a temperature of 40, 43, 45 or 47 ºC for 10 min. After 10 days of incubation, colonies containing more than 50 cells were scored as survivors. In this study, we investigated the effects of the Hsp inhibitor KNK437 [*N*-formyl-3,4-methylenedioxybenzylidene-γ-butyrolactam] [12], which was obtained from Kaneka Corp (Takasago, Japan).

### 5.7. Determination of apoptosis by TUNEL (terminal deoxynucleotidyl transferase nick-end labeling) staining

Detection and quantification of apoptotic cells were carried out using the TUNEL staining method. *In situ* TUNEL staining was carried out using a *In situ* Cell Death Detection kit Fluorescein (Roche Inc., Mannheim, Germany). The heat-treated cells were cultured for 3 days on a chamber slide (Labtek International Lid., Naperville, IL) before TUNEL staining.

### 5.8. Detection of activated p38 MAPK and phospho-Hsp27

p38 MAPK activity was determined as described previously [13]. Briefly, NHDFs were plated at a density of 1 × 10^6^ cells/25 cm^2^ in a flask of serum-containing medium and cultured for 3 days. Then the culture medium was replaced by 0.5% fetal calf serum-containing medium, and the cells were cultured for a further 48 h. Fibroblasts were then exposed to heat shock (temperature of 43 ºC) for 10 min. p38 MAPK activity in cell lysates was then assayed. The cells were lysed in a lysing buffer. Aliquots of the lysates (10–15 μg) from each sample were fractionated on sodium dodecyl sulfate-10% polyacrylamide gel and transferred to polyvinylidene difluoride membranes. The blots were probed with antibodies specific for phospho-Hsp27 and phospho-p38 MAPK (New England BioLabs, Beverly, MA) at a dilution of 1:1,000 in blocking buffer (5% nonfat dry milk) for 12 h at 4 ºC. The blots were probed with a secondary antibody, horseradish peroxidase-linked anti-rabbit immunoglobulin G, at a dilution of 1:2,000 in blocking buffer for 60 min at room temperature. The blots were stained for 1 min using a nucleic acid chemiluminescence reagent (LumiGLO^™^, Kirkegaard and Perry Laboratories Inc., Gaithersburg, MD, USA) and exposed to X-ray film.

### 5.9. Data analysis

Experiments were replicated a minimum of five times, and data were analyzed using one-way analysis of variance. If any significant effect was found, it was assigned to different groups by *post hoc* Bonferroni multiple range tests. Significance was set at *p* < 0.05.
